# RETRACTED: Jabbar et al. Sinapic Acid Attenuate Liver Injury by Modulating Antioxidant Activity and Inflammatory Cytokines in Thioacetamide-Induced Liver Cirrhosis in Rats. *Biomedicines* 2023, *11*, 1447

**DOI:** 10.3390/biomedicines13040862

**Published:** 2025-04-03

**Authors:** Ahmed A. J. Jabbar, Zaenah Zuhair Alamri, Mahmood Ameen Abdulla, Ahmed S. AlRashdi, Soran Kayfi Najmaldin, Mustafa AbdulMonam Zainel

**Affiliations:** 1 Department of Medical Laboratory Technology, Erbil Technical Health and Medical College, Erbil Polytechnic University, Erbil 44001, Iraq; 2 Department of Biological Sciences, Faculty of Science, University of Jeddah, Jeddah 23218, Saudi Arabia; zzalamri@uj.edu.sa; 3 Department of Medical Microbiology, College of Sciences, Cihan University-Erbil, Erbil 44001, Iraq; mahmood.ameen@cihanuniversity.edu.iq; 4 Central Public Health Laboratories, Ministry of Health, P.O. Box 2294, Muscat 111, Oman; ahmed.alrashdi2@moh.gov.om; 5 Department of Medical Analysis, Faculty of Applied Science, Tishk International University, Erbil 44001, Iraq; soran.kayfi@tiu.edu.iq; 6 Department of Pharmacy, College of Pharmacy, Knowledge University, Erbil 44001, Iraq; mustafa.zainel@knu.edu.iq

The journal retracts the article titled “Sinapic Acid Attenuate Liver Injury by Modulating Antioxidant Activity and Inflammatory Cytokines in Thioacetamide-Induced Liver Cirrhosis in Rats” [1], cited above.

Following publication, concerns were brought to the attention of the Editorial Office regarding inappropriate image modification and duplication between this publication [1] and an earlier article [2] published by a similar author group.

Adhering to our complaints procedure, the Editorial Office and Editorial Board conducted an investigation that confirmed unusual image anomalies and duplication between Figure 2E [1] and Figure 1G2 [2], presenting different experimental conditions. Following discussions, the authors were unable to provide satisfactorily explanations, nor appropriate raw material for Editorial Board’s evaluation. Consequently, the Editorial Board has lost confidence in the reliability of the findings and decided to retract this publication [1], as per MDPI’s retraction policy (https://www.mdpi.com/ethics#_bookmark30).

This retraction was approved by the Editor-in-Chief of the journal *Biomedicines*.

The authors did not agree to this retraction.
